# Efficacy and safety of hyperbaric oxygen therapy for diabetes peripheral neuropathy: A systematic review and meta-analysis

**DOI:** 10.1097/MD.0000000000039699

**Published:** 2024-09-06

**Authors:** Jiyan Weng, Haiyong Ren, Qiaofeng Guo, Kai Huang, Liqing Ding

**Affiliations:** a Rehabilitation Department, Tongde Hospital of Zhejiang Province, Hangzhou, China; b Department of Orthopedics, Tongde Hospital of Zhejiang Province, Hangzhou, China; c Department of Endocrinology, Tongde Hospital of Zhejiang Province, Hangzhou, China.

**Keywords:** diabetes peripheral neuropathy, hyperbaric oxygen therapy, meta-analysis, randomized controlled trial

## Abstract

**Background:**

Diabetes peripheral neuropathy (DPN) is mainly treated with diabetes as a whole, and there is no targeted treatment. Some studies have reported that adjuvant hyperbaric oxygen therapy (HBOT) for DPN has achieved a good effect, our study aimed to evaluate the clinical efficacy and safety of HBOT for DPN and provide reference for the clinic by using a systematic review and meta-analysis.

**Methods:**

A comprehensive search was conducted across several databases, including PubMed, Embase, Cochrane Library, Web of Science, China National Knowledge Internet Database, Chinese BioMedical Database, China Scientific Journal Database, and Wanfang Database, for relevant randomized controlled trials published before July 2022. The population, intervention, comparison, outcomes, study design criteria were used to guide the selection of studies. Meta-analysis was performed using RevMan 5.4 and STATA 14.0, with odds ratios and mean differences along with 95% confidence intervals serving as measures of effect size.

**Results:**

Fourteen randomized controlled trials were included in the final analysis, comprising 675 patients in the HBOT group and 648 in the standard therapy (ST) group. The HBOT group demonstrated a significantly higher effective treatment rate compared to the ST group (*P* < .001). Additionally, the HBOT group showed significant improvements in motor nerve conduction velocity (MNCV) and sensory nerve conduction velocity (SNVC) across multiple nerves: median nerve (PMNCV < 0.001, PSNCV = 0.001), ulnar nerve (PMNCV = 0.02, PSNCV < 0.001), peroneal nerve (PMNCV < 0.001, PSNCV < 0.001), and tibial nerve (PMNCV = 0.001, PSNCV = 0.008). Six adverse events were reported in the HBOT group, while no adverse events occurred in the ST group, with no significant difference between the 2 groups. Publication bias was identified in some outcome variables through funnel plots, Begger test, and Egger test.

**Conclusions:**

HBOT significantly enhances treatment efficacy and nerve conduction velocity in patients with DPN, with few adverse events, making it a safe and effective adjunctive therapy for DPN.

## 1. Introduction

Diabetes is a metabolic disease characterized by hyperglycemia resulting from insufficient insulin secretion and insulin resistance. Diabetic peripheral neuropathy (DPN) involves various symptoms and signs of peripheral nerve dysfunction in diabetic patients, occurring when other causes are excluded. These symptoms include the gradual decline and loss of sensory, motor, and autonomic nerves, typically progressing from distal to proximal areas.^[1,2]^ DPN is one of the most common chronic complications of diabetes and a leading cause of disability.^[3]^

Currently, multiple hypotheses exist regarding the pathogenesis of DPN. For instance, the development of DPN is believed to be associated with factors such as microcirculation disturbances, vasoactive agents, oxidative stress, metabolic dysfunction, immune responses, and neurotrophic factors. However, no single mechanism fully explains its pathogenesis.^[4,5]^ The treatment of DPN generally involves the overall management of diabetes, along with the use of vasoactive drugs, neurotrophic agents, antioxidants, and other pharmacological treatments, supplemented by physical therapy and rehabilitation.^[6–9]^ Despite these approaches, traditional medical treatments often yield suboptimal results, offering only limited relief of clinical symptoms for some patients. Although comprehensive rehabilitation measures have improved patients’ quality of life to some extent, their overall effectiveness remains unsatisfactory.

Hyperbaric oxygen therapy (HBOT) has been reported to effectively alleviate nerve tissue hypoxia, promote the early recovery of injured nerves, and reduce clinical symptoms in patients.^[10,11]^ However, some studies have indicated that HBOT does not mitigate nerve damage in animal models of DPN.^[12,13]^ There is currently no effective clinical treatment for DPN, and the use of hyperbaric oxygen therapy remains controversial. Therefore, this study systematically evaluates the efficacy and safety of hyperbaric oxygen therapy for DPN through a meta-analysis, aiming to provide evidence-based guidance for clinical practice and to offer insights for future research.

## 2. Methods

### 2.1. Literature search strategy

A literature search was conducted across PubMed, EMBASE, Cochrane Library, Web of Science, China National Knowledge Infrastructure, Chinese BioMedical Database, China Scientific Journal Database, and Wanfang Database using the following keywords: (1) hyperbaric oxygen therapy, (2) standard therapy, and (3) diabetic peripheral neuropathy. These terms were combined using the Boolean operators “AND” or “OR” to formulate the search strategy. The search was performed without restrictions on language, date, or publication status. Additionally, the reference lists of included studies and relevant review articles were screened to identify further eligible articles for inclusion in our study.

### 2.2. Study selection

Studies meeting the following population, intervention, comparison, outcomes, study design criteria were deemed eligible for inclusion: (P) population: patients with diabetic peripheral neuropathy (DPN). (I) Intervention: the experimental group received adjunctive HBOT in addition to the standard treatment. (C) Comparison: the control group received standard therapy (ST), which could include treatments for blood pressure, blood sugar, and blood lipids, or ST combined with vitamin B group drugs or mecobalamin. (O) Outcomes: efficacy indicators, such as effective rate, motor nerve conduction velocity (MNCV), and sensory nerve conduction velocity (SNCV), as well as safety indicators, including adverse events and side effects. (S) Study design: only randomized controlled trials (RCTs) were included.

### 2.3. Data extraction

Two pairs of reviewers (JyW and HyR) independently screened the titles, abstracts, and full-text articles of potentially eligible studies. Any disagreements were resolved through consensus or with the assistance of a third author. The following data were extracted from the included articles: (1) first author’s name; (2) year of publication; (3) number of patients; (4) gender; (5) age; (6) duration of disease; (7) treatment duration; (8) years since onset; and (9) raw data for variables of interest.

### 2.4. Quality assessment

We assessed the risk of bias for individual elements across 5 domains—selection, performance, attrition, reporting, and other biases—using the Cochrane Collaboration’s tool. The quality scale ranged from 0 to 9, with higher scores indicating better quality. After reviewing the full text, the author assigned a subjective score to each of the 5 domains. Scores of 1 to 3 were considered high risk, 4 to 6 as unclear risk, and 7 to 9 as low risk.

### 2.5. Statistical analysis

The meta-analysis was conducted using either a random-effects or fixed-effects model, depending on the context, with RevMan version 5.4 and STATA version 14.0. Mean difference (MD) was used for continuous variables, odds ratio for binary variables, and 95% confidence intervals (CI) were calculated for both types of measures. Heterogeneity across studies was assessed using the *I*² statistic and Cochran Q test. If *P* > .1 and *I*² < 50%, heterogeneity was considered negligible, and a fixed-effects model was used for analysis; otherwise, a random-effects model was applied to calculate the combined effect. For cases where the number of included studies was ≥10, funnel plots were used for qualitative assessment of publication bias, and Begger test and Egger test were used for quantitative assessment, with *P* < .05 indicating the presence of publication bias.

## 3. Results

### 3.1. Search process

**Figure 1.**
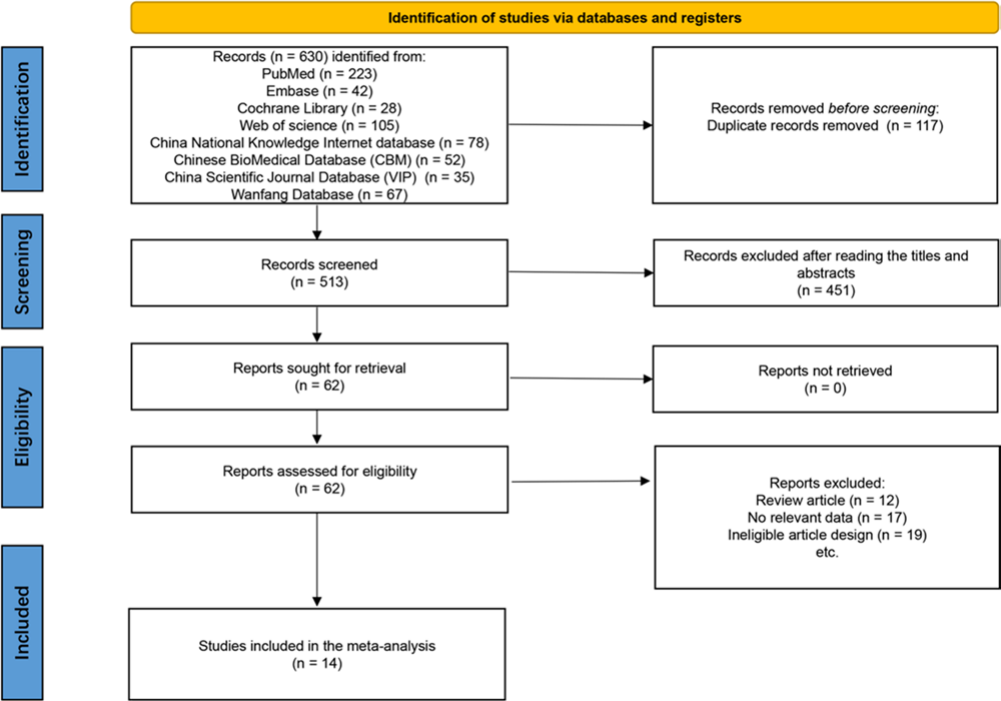
Schematic of the trial selection process.

Figure 1 illustrates the process of screening articles for inclusion in the review and meta-analysis. The search strategy initially identified a total of 630 articles across all databases. After removing duplicates, 513 studies were subjected to title and abstract screening, which narrowed the selection to 62 studies deemed potentially eligible. Following a full-text review and the exclusion of 48 articles, 14 studies were ultimately included in the final analysis.^[14–27]^

### 3.2. Characteristics of the included studies

The baseline characteristics of the patients included in the meta-analysis are detailed in Table 1. The 14 studies, published between 2003 and 2021, comprised 675 patients in the HBOT group and 648 in the ST group. Sample sizes ranged from 41 to 200 patients, all of whom were over 35 years old, with treatment durations ranging from 20 to 30 days.

**Table 1:** Characteristics of included studies.

Study	Study design	Intervention		No. of patients		Gender (M/F)		Age (years)		Course of disease (years)		Treatment time (days)	Years of onset
		Test	Control	Test	Control	Test	Control	Test	Control	Test	Control		
Li 2003^[24]^	RCT	HBOT	ST	21	20	–	–	47.3 ± 5.1	47.3 ± 5.1	2–24	2–24	30	January 2000 to October 2001
Wang 2005^[17]^	RCT	HBOT	ST	38	31	21/17	17/14	52.4 ± 12.5	51.6 ± 10.8	7.1 ± 10.2	6.8 ± 11.2	30	January 1999 to December 2003
Zhao 2005^[25]^	RCT	HBOT	ST	30	30	18/12	20/10	65.7 ± 7.8	65.7 ± 8.2	–	–	30	July 2002 to December 2004
Tan 2006^[14]^	RCT	HBOT	ST	49	38	25/24	23/15	56.7 ± 0.6	55.3 ± 0.8	–	–	40	January 2003 to April 2005
Bai 2009^[16]^	RCT	HBOT	ST	38	38	24/14	18/20	57.1 ± 7.6	59.1 ± 7.7	14.6 ± 4.6	13.9 ± 4.9	40	June 2006 to December 2007
Guan 2009^[23]^	RCT	HBOT	ST	42	36	24/18	20/16	38–76	35–78	1–20	1.2–18	20	–
Chen 2010^[18]^	RCT	HBOT	ST	40	40	22/18	26/14	56 ± 5	54 ± 6	–	–	30	–
Han 2010^[22]^	RCT	HBOT	ST	21	23	9/12	10/13	38–76	35–78	1–20	1.2–18	20	March 2008 to December 2009
Jin 2010^[21]^	RCT	HBOT	ST	56	52	30/26	28/24	58 ± 10	56 ± 9	8.6 ± 3.2	8.6 ± 3.4	28	–
Tang 2012^[27]^	RCT	HBOT	ST	71	71	37/34	39/31	57.4 ± 11.9	58.1 ± 12.2	12.1 ± 4.1	11.3 ± 4.7	28	June 2005 to May 2011
Tong 2013^[20]^	RCT	HBOT	ST	20	20	–	–	45–75	45–75	0.5–16	0.5–16	28	February 2010 to February 2011
Chang 2017^[19]^	RCT	HBOT	ST	99	99	65/34	60/39	61.2 ± 3.4	63.2 ± 4.4	10.2 ± 2.5	10.3 ± 2.6	24	September 2015 to September 2016
Zhang 2019^[26]^	RCT	HBOT	ST	100	100	51/49	52/48	66.5 ± 3.0	65.5 ± 3.5	3.0 ± 2.0	3.0 ± 2.0	30	February 2010 to February 2016
Li 2021^[15]^	RCT	HBOT	ST	50	50	27/23	25/25	70.8 ± 10.7	71.8 ± 10.9	15.5 ± 2.7	15.2 ± 3.1	30	December 2018 to December 2019

HBOT = hyperbaric oxygen therapy, RCT = randomized controlled trial, ST = standard therapy.

### 3.3. Results of quality assessment

**Figure 2.**
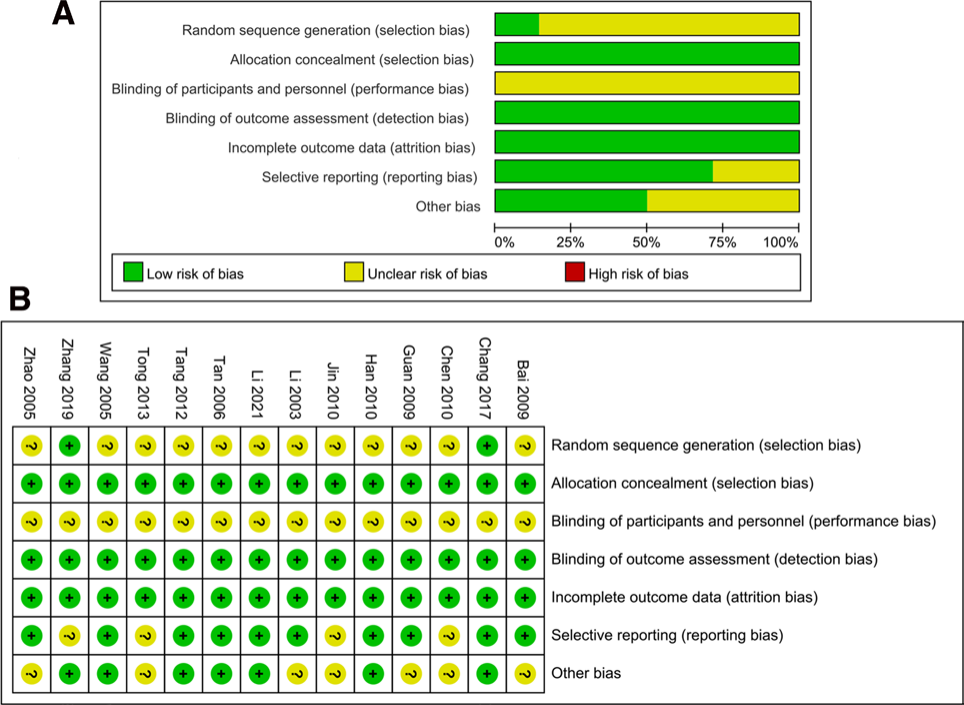
Risk of bias of included studies: low (green), unclear (yellow), and high (red). (A) Summary of bias assessments, (B) risk of bias for each study.

The Cochrane risk of bias assessment tool was utilized to evaluate the methodological quality of the 14 trials. While all studies employed randomization, only 2 explicitly mentioned the use of random number generation. None of the studies reported using double-blinding, which may indicate performance bias. Four studies reported fewer outcome variables, suggesting potential reporting bias. Additionally, the baseline data in 7 studies did not address comparability, indicating the possibility of other biases. A summary of the risk of bias assessment for the included studies is presented in Figure 2.

### 3.4. Results of meta-analysis

#### 3.4.1. Effective rate

**Figure 3.**
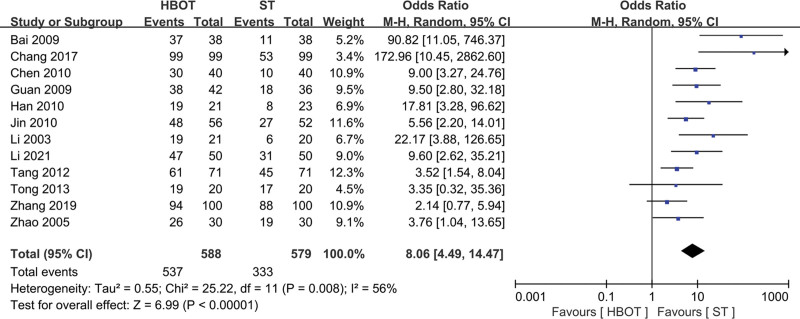
Forest plot for selected studies reporting effective rate. HBOT = hyperbaric oxygen therapy; ST = standard therapy.

The pooled analysis showed that the HBOT group had a significantly higher effective rate compared to the ST group, with an odds ratio of 8.06 (95% CI: 4.49 to 14.47, *P* < .00001, random-effects model) (Fig. 3). However, moderate heterogeneity was observed among the studies (*I*² = 56%, *P* = .008).

### 3.5. Motor nerve conduction velocity

**Figure 4.**
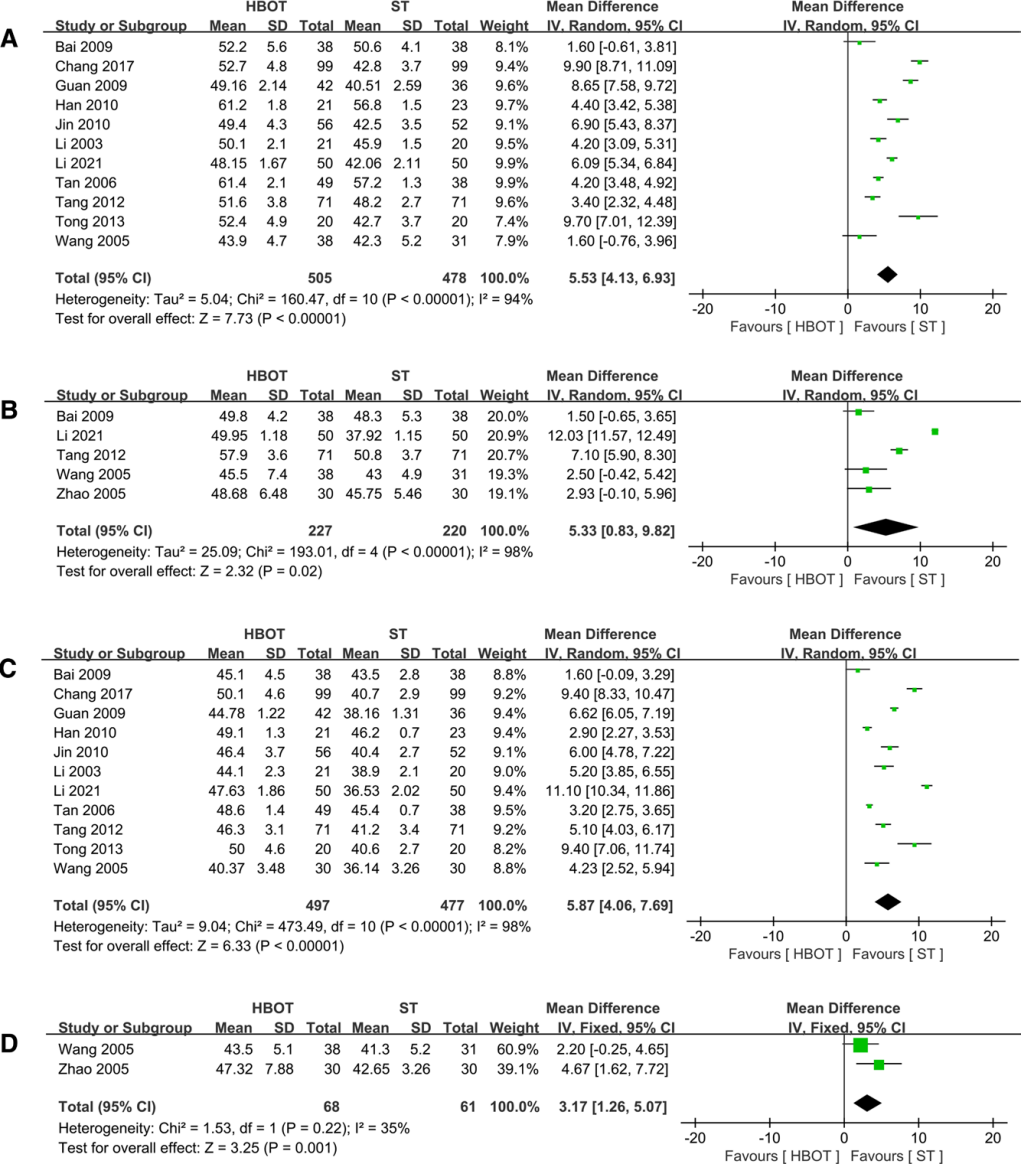
Forest plot for selected studies reporting MNCV. (A) Median nerve; (B) ulnar nerve; (C) peroneal nerve; (D) tibial nerve. HBOT = hyperbaric oxygen therapy; MNCV = motor nerve conduction velocity; ST = standard therapy.

A meta-analysis was conducted on studies examining the MNCV of the median, ulnar, peroneal, and tibial nerves, with 11, 5, 11, and 2 articles included in the analysis, respectively. Except for the meta-analysis of the tibial nerve, which showed low heterogeneity and was analyzed using a fixed-effects model (*P* = .22), the other 3 analyses demonstrated significant heterogeneity and were assessed using a random-effects model (*P* < .00001). The results indicated that the HBOT group significantly improved MNCV in the median nerve (MD = 5.53, 95% CI 4.13 to 6.93, *P* < .00001, Fig. 4A), ulnar nerve (MD = 5.33, 95% CI 0.83 to 9.82, *P* = .02, Fig. 4B), peroneal nerve (MD = 5.87, 95% CI 4.06 to 7.69, *P* < .00001, Fig. 4C), and tibial nerve (MD = 3.17, 95% CI 1.26 to 5.07, *P* = .001, Fig. 4D) compared to the ST group.

### 3.6. Sensory nerve conduction velocity

**Figure 5.**
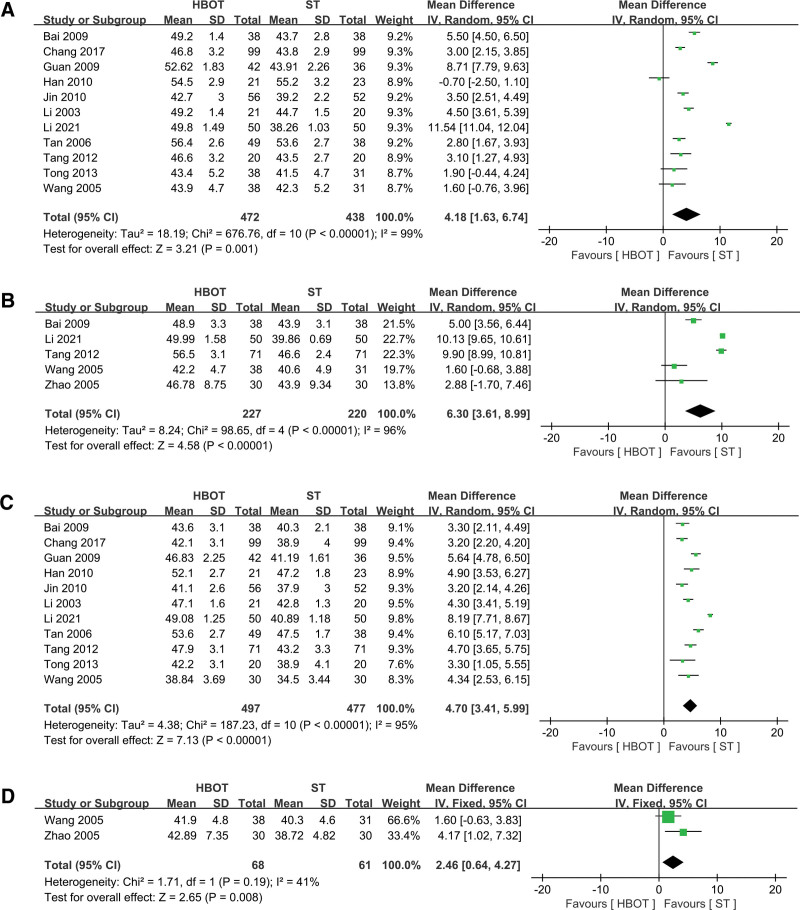
Forest plot for selected studies reporting SNCV. (A) Median nerve; (B) ulnar nerve; (C) peroneal nerve; (D) tibial nerve. HBOT = hyperbaric oxygen therapy; SNCV = sensory nerve conduction velocity; ST = standard therapy.

Similarly, we conducted a meta-analysis of studies examining SNCV in the median, ulnar, peroneal, and tibial nerves, with 10, 5, 11, and 2 articles included, respectively. The heterogeneity tests for the meta-analyses of the median, ulnar, and peroneal nerves were statistically significant (*P* < .00001), while the heterogeneity for the tibial nerve meta-analysis was lower (*P* = .19). The results indicated that, compared to the ST group, the HBOT group significantly improved SNCV in the median nerve (MD = 4.18, 95% CI 1.63 to 6.74, *P* = .0001, Fig. 5A), ulnar nerve (MD = 6.30, 95% CI 3.61 to 8.99, *P* < .00001, Fig. 5B), peroneal nerve (MD = 4.70, 95% CI 3.41 to 5.99, *P* < .00001, Fig. 5C), and tibial nerve (MD = 2.46, 95% CI 0.64 to 4.27, *P* = .008, Fig. 5D).

### 3.7. Adverse events

A total of 391 patients across 4 studies reported adverse events. In the HBOT group, 6 out of 201 patients experienced adverse events, including symptoms of earache, tinnitus, and vertigo, while none of the 190 patients in the ST group reported any adverse events. The difference between the 2 groups was not statistically significant (*P* > .05).

### 3.8. Publication bias

**Figure 6.**
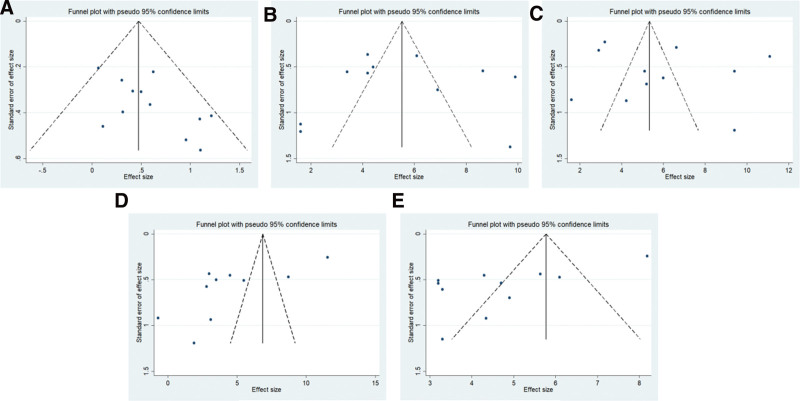
Funnel plot of publication bias risk. (A) Effective rate; (B) MNCV of median nerve; (C) MNCV of peroneal nerve; (D) SNCV of median nerve; (E) SNCV of peroneal nerve. MNCV = motor nerve conduction velocity; SNCV = sensory nerve conduction velocity.

When the number of included studies for a given indicator was ≥10, it was necessary to assess publication bias. We qualitatively examined publication bias by evaluating the symmetry of funnel plots and quantitatively assessed it using Begger and Egger tests. In our study, the number of included studies for effective rate, MNCV of the median nerve, MNCV of the peroneal nerve, SNCV of the median nerve, and SNCV of the peroneal nerve all exceeded 10. The funnel plots for these 5 indicators are shown in Figure 6, and the results of Begger and Egger tests are presented in Table 2. The funnel plots for these indicators did not appear symmetrical. Although the *P* values from Begger test were >.05, the *P* values from Egger test for effective rate, SNCV of the median nerve, and SNCV of the peroneal nerve were <.05, indicating the presence of publication bias in the included studies for these 3 indicators.

**Table 2:** Publication bias by the Egger test and the Begger test.

Outcomes indicators	No. of studies	Egger’s test *P*	Begger test *P*
Effective rate	12	.041	.115
*MNCV*			
Median nerve	11	.901	.533
Peroneal nerve	11	.458	.876
*SNCV*			
Median nerve	10	.009	.592
Peroneal nerve	11	.004	.350

MNCV = motor nerve conduction velocity, SNCV = sensory nerve conduction velocity.

## 4. Discussion

HBOT can enhance oxygen supply to body tissues, improve oxygen diffusion distances, promote neovascularization, boost the bactericidal efficacy of antibiotics, and support nerve function recovery. Although HBOT has the potential to alleviate DPN symptoms in theory, its effectiveness as a routine clinical treatment remains uncertain.^[28,29]^

This study ultimately included 14 RCTs that met strict inclusion criteria. The baseline characteristics of each study population were balanced, and there was no indication of allocation concealment. However, none of the studies provided a detailed explanation of blinding methods. Some studies reported fewer outcome variables, but the overall research quality was deemed acceptable. The heterogeneity test revealed significant heterogeneity across multiple outcome variables, and the funnel plots showed asymmetry. Additionally, Egger test indicated some publication bias, suggesting that the overall strength of the evidence in this study is moderate.

In our study, 675 patients were included in the HBOT group and 648 in the ST group. The meta-analysis demonstrated that, in terms of effectiveness, the HBOT group significantly improved the effective rate for DPN patients compared to the ST group. Regarding nerve conduction velocity, the HBOT group showed significantly better results in both MNCV and SNCV after treatment compared to the ST group. As for safety, only one study reported adverse reactions in 6 patients, who experienced symptoms such as earache, tinnitus, and dizziness, while no adverse events were reported in the ST group; however, there was no statistically significant difference between the 2 groups. Huang and colleagues conducted a similar meta-analysis, and their findings were consistent with ours.^[30]^ Sharma study evaluated the role of HBOT in the treatment of diabetic foot ulcers and found that HBOT was effective as an adjunctive therapy. The results suggested that HBOT can promote ulcer healing and reduce the incidence of amputation in patients with diabetic foot ulcers.^[31,32]^

Diabetic foot ulcers are lower limb infections and ulcers that occur in diabetic patients with neuropathy and peripheral vascular disease. Sharma research further supports the efficacy of HBOT in treating DPN, highlighting its effectiveness in managing related complications.

Nerve conduction velocity is widely recognized as the “gold standard” for the electrophysiological assessment of DPN.^[33]^ It primarily detects changes in SNCV and MNCV in myelinated large nerve fibers, including the median, ulnar, peroneal, and tibial nerves.^[34]^ Our study suggests that HBOT can effectively improve both SNCV and MNCV. This improvement is likely due to HBOT’s ability to increase blood oxygen concentration and rapidly elevate the amount of oxygen dissolved in plasma to levels sufficient for tissue utilization, thereby reducing the reliance on oxygen carried by hemoglobin and enhancing the oxygen supply to various organs.^[35]^ Additionally, nerve growth factors are proteins that promote the survival, morphological development, and functional differentiation of nerve cells.^[36]^ They play a crucial role in delaying the onset and progression of DPN. Neurotrophic factors and their receptors, which are widely distributed throughout the central and peripheral nervous systems, contribute to the recovery of motor and sensory functions following nerve injury. Hyperbaric oxygen therapy can activate the expression of protective and growth-promoting factors in tissue cells, enhance the synthesis of molecular chaperone proteins, improve vascular function, and mitigate damage to peripheral blood vessels.^[37]^ Additionally, HBOT can induce and enhance the expression of nitric oxide synthase in endothelial cells, increase the synthesis of skin-derived nitrogen oxides in microvessels, relax microvessels, improve microcirculation, reduce peripheral nerve damage, and provide a protective effect.^[38]^

This meta-analysis has certain limitations. Firstly, all the included studies were conducted in China, and details regarding randomization and blinding methods were not thoroughly explained, which may limit the quality of the included studies in this evaluation. The extrapolation of results is also constrained by regional factors. Additionally, there was significant heterogeneity among the studies, but it was not possible to perform further subgroup analyses based on clinical experience, which may affect the strength of the evidence supporting the conclusions.

## 5. Conclusions

In summary, HBOT has shown better efficacy in treating patients with DPN, significantly improving the effective rate and enhancing the conduction function of peripheral nerves, with fewer adverse reactions and a high safety profile. However, due to the quality of the included studies, these conclusions require further validation through high-quality and rigorously designed RCTs to provide more robust scientific evidence for the clinical application of HBOT.

## Author contributions

**Conceptualization:** Liqing Ding.

**Data curation:** Kai Huang.

**Investigation:** Haiyong Ren.

**Methodology:** Haiyong Ren.

**Software:** Qiaofeng Guo.

**Writing – original draft:** Jiyan Weng, Qiaofeng Guo.

**Writing – review & editing:** Liqing Ding.
